# A systematic review of rye (*Secale cereale* L.) as a source of resistance to pathogens and pests in wheat (*Triticum aestivum* L.)

**DOI:** 10.1186/s41065-017-0033-5

**Published:** 2017-05-25

**Authors:** Leonardo A. Crespo-Herrera, Larisa Garkava-Gustavsson, Inger Åhman

**Affiliations:** 1 International Maize and Wheat Improvement Center (CIMMYT), Apdo. Postal 6-641, 06600 Mexico, DF Mexico; 2 Department of Plant Breeding, Swedish University of Agricultural Sciences, P.O. Box 101, SE 23053 Alnarp, Sweden

**Keywords:** Wheat breeding, Disease, Insect, Mite, Nematode, Substitution, Translocation

## Abstract

Wheat is globally one of the most important crops. With the current human population growth rate, there is an increasing need to raise wheat productivity by means of plant breeding, along with development of more efficient and sustainable agricultural systems. Damage by pathogens and pests, in combination with adverse climate effects, need to be counteracted by incorporating new germplasm that makes wheat more resistant/tolerant to such stress factors. Rye has been used as a source for improved resistance to pathogens and pests in wheat during more than 50 years. With new devastating stem and yellow rust pathotypes invading wheat at large acreage globally, along with new biotypes of pest insects, there is renewed interest in using rye as a source of resistance.

Currently the proportion of wheat cultivars with rye chromatin varies between countries, with examples of up to 34%. There is mainly one rye source, Petkus, that has been widely exploited and that has contributed considerably to raise yields and increase disease resistance in wheat. Successively, the multiple disease resistances conferred by this source has been overcome by new pathotypes of leaf rust, yellow rust, stem rust and powdery mildew. However, there are several other rye sources reported to make wheat more resistant to various biotic constraints when their rye chromatin has been transferred to wheat. There is also development of knowledge on how to produce new rye translocation, substitution and addition lines. Here we compile information that may facilitate decision making for wheat breeders aiming to transfer resistance to biotic constraints from rye to elite wheat germplasm.

## Background

### The use of rye chromatin in wheat

Wheat (*Triticum aestivum* L.) provides about 20% of the calories in the diet of the human population. Augmenting its productivity is a global task of paramount importance. The demand for this crop is increasing at a higher rate than its production, and at the same time there is also a great need for producing it without compromising the environment. Plant breeding, in combination with environmentally friendly production systems with increased efficiency are required to meet the demands [1].

Wheat yields are constrained by several stresses, biotic and abiotic. To counteract them, plant breeders continuously try to incorporate new resistance/tolerance traits in new cultivars by making use of the inherent diversity of domesticated wheat and also of its related species. One of the most widely used wheat relatives in this effort is rye (*Secale cereale* L.). Genes from rye have been incorporated in wheat in the form of substitution and translocation lines. Indeed, several studies show chromosome 1R to contribute a yield advantage in wheat (e.g. [2–4]).

The first attempts to hybridize wheat and rye can be traced back to the experiments conducted by Stephen Wilson, presented in 1873 [5]. Although he considered his results to be negative, he stated that the failure could have been caused by improper methods. The first stable amphiploid triticale (*Triticosecale* Wittmack) is attributed to Rimpau in 1888, and thereafter more efforts were put into producing wheat-rye hybrids. The advent of colchicine treatment and tissue culture at the beginning of the twentieth century greatly facilitated the production of triticales [6].

Historically, mainly four rye sources have been used to incorporate rye chromatin in wheat, deployed as (1B)1R substitution or 1BL.1RS and 1AL.1RS translocation lines. The first and most widely deployed source is 1R from Petkus rye. Genotypes with 1BL.1RS translocations were first developed in Germany by Riebesel via extensive crossings between wheat and rye, and such a translocation from Petkus became ancestor of wheat cultivars released in Western Europe, Russia, Mexico, Chile and other countries. From triticale, it is possible to develop wheat germplasm with chromosomes or chromosome arms exchanged for rye chromatin. The other three historic sources for cultivar development were of that kind, developed in Japan, Germany and USA [7].

Between 1960 and 1990, several hundreds of cultivars with (1B)1R substitution, or 1BL.1RS and 1AL.1RS translocations were released. At the International Maize and Wheat Improvement Center (CIMMYT), 60% of the wheat descendants were 1BL.1RS genotypes during the 1990’s [7]. In China, about 40% of the wheat cultivars released between 1960 and 2000 were 1B/1R translocations with yield gains over the years partly attributed to this characteristic [4]. There are no recent surveys published on the proportion of wheat cultivars with rye chromatin. However, Schlegel [8] has compiled a worldwide list of 2470 wheat cultivars and experimental lines that carry alien introgressions. According to such information and the cultivar listing by the International Union for the Protection of New Varieties of Plants [9], we could estimate that there are countries such as Chile in which 34% of the commercial varieties released between 2000 and 2013 carry rye introgressions. In other UPOV countries this percentage is as low as 1–2%, for instance in Russia and Australia. In the USA, according to the database of the Journal of Plant Registrations (http://www.ars-grin.gov/cgi-bin/npgs/pvp/pvplist.pl?) and Schlegel’s compilation, the percentage of commercial varieties carrying rye chromatin is about 15%. These examples demonstrate how the current importance of rye introgressions in wheat varies between countries.

Partly due to new pathotypes of stem and yellow rusts invading wheat at large scale, in Africa, northern Europe and China, there is renewed interest in using rye as a source for new resistance genes in wheat. Here we compile historic and more recent information on resistance to biotic stresses transferred from rye to wheat.

## Transfer of rye chromatin to wheat

Wheat, *T. aestivum* (2n=6x=42), originates from the Near East. It is composed of three genomes (A, B and D) from three diploid ancestors. Donors of the A and D genomes are relatives of *Triticum urartu* Tumanian ex Gandilyan and *Aegilops tauschii* Coss., respectively. The origin of the B genome is not completely clarified, but certain evidence points out *Aegilops speltoides* Tausch as the relative of the donor. Hexaploid wheat originates from the hybridization of *Triticum turgidum* L. (AABB) and *A. taushii* (DD) relatives. Despite its polyploid nature, wheat shows a diploid-like behavior with preferential pairing between homologous chromosomes during meiosis [10–12].

Rye, *S. cereale* (2n=2x=14), is a diploid species that also originates from the Near East [13]. The chromosome groups 1, 2, 3, 5 and 6 of wheat are essentially homoelogous with 1R, 2R, 3R, 5R and 6R chromosomes of rye, and 4 and 7 of wheat have partial reciprocal homoeology with groups 4R and 7R [14].

The allopolyploid nature of wheat makes it highly tolerant to modifications in its genetic composition. The homoeologous pairing between rye and wheat allows the introduction of desirable agronomic characteristics in wheat from rye such as resistance to certain pests and diseases and tolerance to various abiotic stresses. Additionally, the buffering capability of wheat for tolerating important modifications in its genome has allowed the development of different genetic stocks consisting of monosomic, telocentric, deletion and nullisomic lines. These types of plant material have played a significant role in genetic research, for instance in the determination of physical locations for various molecular markers and genes [15, 16].

The transfer of a target chromosome to be incorporated in wheat can be done by selecting an adequate aneuploid wheat line and cross it with rye or a previously developed amphiploid, in this particular case triticale. Wheat-alien substitution lines are frequently used as bridges to produce wheat-alien translocation lines [10, 16, 17].

Another strategy to obtain translocation lines is to recover spontaneous wheat-rye translocations that occur due to the centromeric breakage and fusion of the chromosome arms. Chromosomes tend to break at the centromeres during meiotic metaphase I forming telocentrics. Different telocentrics may fuse again, and thereby exchange chromosome segments [17, 18].

Inducing random translocations by irradiation methods in the absence of homoeologous pairing of substitution lines is yet another strategy, although this method can be laborious and cause several deleterious effects [19]. The winter wheat cultivar Amigo with the 1AL.1RS translocation was developed with this method [20], although doubts have been expressed whether this centric translocation was caused by the irradiation treatment [16].

It is also possible to obtain translocation lines with the procedure described by Lapitan et al. [21], in which the embryos of the wheat-rye hybrids are grown in tissue culture on a medium with enhanced auxin concentration to stimulate callus formation. Recovered plants are colchicine treated for chromosome doubling. With this procedure the generation of translocation lines is facilitated by the tissue culture step in which different structural changes in the chromosomes occur. Lines produced with this method were identified to carry 4DL.1RS, 2BS.2RL and 2BL.3R translocations [21, 22].

Another strategy is by using a *ph1b* mutant of wheat. The functional *Ph1* allele in chromosome 5B inhibits homoeologous pairing between wheat and alien chromosomes whereas the mutant allows pairing and recombination between homoeologues. Thus it is possible to reduce the amount of genetic material introduced from the alien species [10, 17]. However translocation break points between homoeologous chromosomes are mostly concentrated to the distal parts of the chromosomes [23].

Yet another strategy to induce homoeologous pairing between rye and wheat chromosomes is to expose tillers to okadaic acid before cells enter into meiosis phase. This will induce early condensation of chromatin, which is associated with the phenotype of *ph1* mutants. When applying optimal concentrations of okadaic acid, homoeologous pairing can take place even in the presence of the *Ph1* allele [24].

Many Chinese wheat cultivars have *Kr* alleles which make them easily crossable with rye, something which has been used in more recent efforts to create new 1BL.1RS translocation lines [25, 26].

## Rye as a source of resistance to biotic stresses in wheat

Rye is well documented as a rich source of resistance to pests and pathogens in wheat. Most of its desirable characteristics have been found in chromosome 1R. Nonetheless, resistance is conferred to wheat from the incorporation of other rye chromosomes as well. However, we found no reports on this from chromosome 7R and just one report, on aphid resistance, from chromosome 5R [27]. One advantage of transferring rye chromatin into wheat is that if multiple resistances to various diseases/pests are present in the rye chromosome of interest, the rye chromatin is inherited as a block when crossing with wheat. On the other hand it can be disadvantageous if the rye source confers undesirable traits, and thus makes it necessary to reduce the alien segments.

### Resistance to diseases

Various rye sources incorporated in wheat have been reported to confer resistance to leaf rust (*Puccinia triticina* Erikss.), yellow rust (*Puccinia striiformis* var. *striiformis* Westend), stem rust (*Puccinia graminis* Pers. f. sp. *tritici* Erikss. and E. Henn.), and powdery mildew (*Blumeria graminis* [DC.] f. sp. *tritici* Em. Marchal) (Table 1).

**Table 1:** Resistance to biotic stresses transferred from rye into wheat

	Rye chromosome								
	**1R**			**2R**			**3R**		
Stress	Gene^a^	Description	Germplasm	Gene	Description	Germplasm	Gene	Description	Germplasm
*Puccinia triticina*	*Lr26*	1BL.1RS	Petkus rye; Kavkaz and Veery wheat derives [65, 66]	*Lr25*	4BS.4BL-2RL	Rosen rye; Transec [65, 67]			
				*Lr45*	2AS-2RS.2RL	Petkus rye; RL6144 [65, 68]			
				ND	2BS.2RL	2BS.2RL-SLU [69]			
*Puccinia striiformis* var. *striiformis*	*Yr9*	1BL.1RS	Petkus rye; Kavkaz and Veery wheat derives [65, 70]						
	*YrCn17*	1BL.1RS	Petkus rye L155; R14, Chuan-nong 17 [71, 72]						
	*YrR212*	1BL.1RS	Dwarf rye R12; R212 [71]						
	*yrCH45-1* ^*b*^	1BL.1RS	SW1862; Chuanmai45 [73]						
	ND	1BL.1RS	Aigan rye [74]						
*Puccinia graminis* f. sp. *tritici*	*Sr31*	1BL.1RS	Petkus rye; Kavkaz and Veery wheat derives [65]	*Sr59*	2DS.2RL	Triticale VT828041 [75]	*Sr27*	3AL.3RS	Imperial rye; WRT238 [65, 76]
	*Sr50/SrR*	1DL.1RS	Imperial rye [70]	ND	2BS.2RL	2BS.2RL-SLU [69]			
	*Sr1RS* ^*Amigo*^	1AL.1RS	Amigo wheat [65]						
*Blumeria graminis* f. sp. *tritici*	*Pm8*	1BL.1RS	Petkus rye; Kavkaz and Veery wheat derives [65, 77]	*Pm7*	4BS.4BL-2RL	Rosen rye; Transec [65]			
	*Pm17*; allelic to *Pm8*	1AL.1RS	Insave rye; Amigo wheat derives [65, 77]	ND	2BS.2RL	2BS.2RL-SLU [69]			
	*PmCn17*	1BL.1RS	Petkus rye L155; R14, Chuan-nong 17 [72]	ND	(2D)2R	German white rye; WR02-145 [78]			
				*PmJZHM2RL* ^*b*^	(1D)1R+2R	Jingzhouheimai rye; H-J DA2RDS1R(1D) [79]			
*Schizaphis graminum*	*Gb2*	1AL.1RS	Insave rye; Amigo wheat derives [20, 41, 65]						
	*Gb6*	1AL.1RS	Insave rye; GRS1201 [41, 65, 80, 81]						
*Diuraphis noxia*	*Dn7*	1BL.1RS	Turkey 77 rye; 94M370 wheat [65, 82, 83]						
*Rhopalosipum padi*	ND	1AL.1RS	Panda triticale; E12165 wheat [47, 84]						
	ND	(1D) 1R	Presto triticale [49, 84]						
*Sitobion avenae*	ND	1AL.1RS	Different sources: E12165 wheat, Amigo wheat, Rhino triticale [47, 84]						
	ND	(1D) 1R	Presto triticale [49, 84]						
*Mayetiola destructor*				*H21*	2BS.2RL	Chaupon rye; KS85HF 011-5 [22, 65]			
*Aceria tosichell*	*Cmc3*	1AL.1RS	Insave rye; Amigo wheat [85]						
*Heterodera avenae* *Heterodera filipjevi*									
	Rye chromosome								
	**4R**			**6R**					
Stress	Gene	Description	Germplasm	Gene	Description	Germplasm			
*Puccinia triticina*									
*Puccinia striiformis* var. *striiformis*									
*Puccinia graminis* f. sp. *tritici*									
*Blumeria graminis*	ND	4BL.4RL + 7AS.4RS	German white rye; WR41-1 [86]	*Pm20*	6BS.6RL	Prolific rye; WGRC28 [65]			
	ND	5DS-4RS.4RL	Kustro rye and MK25 triticale [87]	ND	6RL	Kustro rye and triticale MK25 [87]			
				ND	6R	German white rye; WR49-1 [88]			
*Schizaphis graminum*									
*Diurarphis noxia*									
*Rhopalosiphum padi*									
*Sitobion avenae*									
*Mayetiola destructor*				*H25*	4BS.4BL-6RL	Balbo rye; 88HF16 wheat [65, 89]			
*Aceria tosichell*									
*Heterodera avenae*				*CreR*	6DS.6RL	T-701 triticale derives [90]			
*Heterodera filipjevi*				ND	(6D) 6R	[91]			

^a^Gene designations according to McIntosh et al. [26] unless: ^b^additional with temporary designation or *ND* no designation

Chromosome 1R from Petkus rye has been the most deployed of the rye resistance sources over the years since the 1960’s. This has conferred resistance to several important diseases of wheat. It carries *Lr26*, *Yr9*, *Sr31* and *Pm8* resistance genes for leaf rust, yellow rust, stem rust and powdery mildew, respectively. Unfortunately, diseases are able to overcome major race-specific resistance genes like these. However, one remarkable case is the resistance gene *Sr31* from Petkus rye that remained effective against stem rust for more than 30 years. When found defeated first in Uganda in 1999 [28], this posed a major threat to global wheat production because a great proportion of cultivars worldwide carried this gene [29].

Apart from Petkus rye and the wheat cultivars with the 1BL.1RS translocation derived from this source, like Kavkaz and Veery, there are several other 1R sources of resistance to diseases. Insave rye deployed as 1AL.1RS chromatin in Amigo wheat carry stem rust resistance gene *Sr1RS* ^*Amigo*^ and powdery mildew resistance gene *Pm17*, allelic to *Pm8*, and Imperial rye provides stem rust resistance gene *Sr50/SrR*. In China there are many recent attempts to transfer new yellow rust resistance from rye sources, such as that from dwarf rye R12 with temporary gene designation *YrR212*, a 1BL.1RS translocation giving recessive yellow rust resistance from the 1BL.1RS source SW1862 and Aigan rye contributing a non-designated *YR* gene. Also in China, there are now breeding lines and cultivars with 1RS derived from Petkus rye but with other alleles than *Yr9* and *Pm8*, temporarily designated as *YrCn17* and *PmCn17*. Since rye is out-crossing, there may be such within-cultivar allelic variation [25] (Table 1).

There are less cases of disease resistance in source chromosomes other than 1R. However, from 2R there are two designated genes for leaf rust resistance, *Lr25* and *Lr45*, and one stem rust resistance gene, *Sr59*, giving resistance to many stem rust races including Ug99. The 2BS.2RL-SLU source gives resistance to leaf rust, stem rust and powdery mildew. In 2R introgressions there are three more cases of powdery mildew resistance, one of which has gene designation *Pm7. Lr25* and *Pm7* are derived from the same rye source, Rosen. In 3R only the gene *Sr27* for stem rust resistance has been reported so far, from the rye cultivar Imperial. Furthermore, there is powdery mildew resistance in 4R and 6R, from Kustro and German white rye, and in 6R from Prolific rye with gene designation *Pm20* (Table 1). Rahmatov et al. [30] investigated a large set of rye introgression lines in spring and winter wheat for resistance to several virulent races of stem rust and found (1D) 1R, (2D) 2R, (3D) 3R substitution or translocation lines likely to carry new resistance genes. The same large set of rye introgression lines was also tested for yellow rust resistance, with promising results in terms of resistance from hitherto unexploited triticale origin [31].

Even though rye chromatin with resistance genes is present, such genes may not be expressed due to the presence of suppressors in wheat. There are leaf rust resistance suppressors reported in the three genomes of hexaploid wheat [32]. Suppression of the powdery mildew resistance gene *Pm8* is reported to be associated with *Pm3* alleles located in the 1A chromosome. Initially the hypothesis was that the gliadin loci *Gli-A1* and *Gli-A3* were suppressing *Pm8*. Later, when the gene *Pm3* was cloned and shown to be closely linked to the gliadin locus, its role in *Pm8* suppression became evident as a post-translational process [33–37].

The gene *Pm8* has been reported to give different virulence/avirulence patterns in different countries. For instance in Hungary it appears to be ineffective [38] whereas in Norway it is more effective than in China [39].

### Resistance to pests

There are several examples where genes from rye confer resistance to some of the most important wheat pests; like the aphids *Schizaphis graminum* (Rondani), *Diuraphis noxia* (Mordvilko), *Rhopalosiphum padi* L. and *Sitobion avenae* (F.); the cecidomyid *Mayetiola destructor* (Say); the nematodes *Heterodera avenae* (Wollenweber) and *Heterodera filipjevi* (Madzhidov) Stelter; and the mite *Aceria tosichell* Keifer (Table 1).

One of the first reports of transferring resistance to insects from rye into wheat is the resistance gene *Gb2* effective against certain biotypes of *S. graminum.* This gene originates from the chromosome 1RS of Insave rye and is present in the winter wheat cultivar Amigo. The *Gb2* gene confers resistance to biotypes B, C and J of *S. graminum*. However, likely due to the presence of high *S. graminum* genetic diversity in nature, the *Gb2* resistance gene became ineffective in cultivated wheat. Another resistance gene was reported later, *Gb6*, which also originates from the 1RS chromosome arm of Insave rye and this is in addition effective against biotypes E, G, I and K. The *S. graminum* biotypes E and I are currently the most commonly found biotypes in wheat crops in the USA and cause a greater yield loss than the other biotypes [40–42].

Another important example of insect resistance from rye is the gene *Dn7* for *D. noxia* resistance. This gene originates from the 1RS chromosome arm of Turkey 77 rye. In the USA, *D. noxia* was first found in 1986, and only one biotype prevailed until 2003 when a new biotype designated as biotype 2 appeared in wheat [43]. Only *Dn7* is effective to biotype 2 of all the known *Dn* genes. Many additional *D. noxia* biotypes have been found since then, and presently there is no known effective resistance in wheat to biotype 3 of *D. noxia* [44, 45]. Resistance to *D. noxia* in Syria was localized to rye introgressions (1D) 1R, 3DL.3RS and (5D) 5R, but so far without gene designations [27].

Centromeric breakage-fusion and the utilization of *ph1* mutants were exploited by Lukaszewski [23, 46–50], Lukaszewski et al. [51] and Zhang et al. [52] to produce and analyze various substitution, translocation and recombinant lines from different sources of rye in the background of the spring wheat cultivar Pavon F76. Out of this set of 61 lines, certain 1R or 1RS lines from two triticale sources, Panda and Presto, show seedling resistance to both of aphid species *R. padi* and *S. avenae*. It is not known as yet whether resistance is conferred by the same or different rye genes. One line with 1RS from Amigo wheat was resistant to *S. avenae* both at seedling and adult plant stage.

Another rye gene in Amigo wheat, *Cmc3* in 1AL.1RS, gives resistance to the mite *A. tosichell*. Furthermore, resistance to the cecidomyid *M. destructor* has been found in 2RL and 6RL and gene designations have been made as *H21* and *H25*, respectively. Resistance to the nematodes *H. filipjevi* and *H. avenae* has been reported in 6R wheat derives (Table 1).

## Examples of other traits affected by rye chromatin in wheat

Depending on the wheat genetic background, the rye source and the type of abiotic stress factors, studies have shown that rye transferred into wheat may have both positive and negative effects on wheat performance. Hoffman [53] and Waines and Ehdaie [54] have reported 1RS to promote root biomass growth. Karki et al. [55] concluded that the 1BL.1RS translocation is more suited to withstand moisture limitations compared to 1AL.1RS or 1DL.1RS. However, Monneveux et al. [56] have reported that 1BL.1RS negatively impacts yield when wheat is grown under rainfed conditions and heat stress, depending on the wheat background. It is possible that these differences in performance may be due to the presence of suppressors in wheat as is the case in relation to certain diseases.

Kim et al. [3] tested different rye sources of 1R in the genetic background of the spring wheat cultivar Pavon F76. They concluded that it is important to consider which 1RS source is to be transferred into wheat, whereas a favourable 1RS confers higher yield regardless of which wheat chromosome, 1A, 1B or 1D, it is translocated into. However, the position of 1RS in the wheat genome can negatively affect baking quality, and genotypes 1AL.1RS are preferred over 1BL.1RS and 1DL.1RS in this respect [57].

Additional traits that can potentially be exploited are aluminum and acid soil tolerance. It is known that rye possesses tolerance to these soil conditions in chromosomes 3R, 4R and 6R. However, presence of gene suppressors in the wheat genome may hamper full expression of these traits [58]. Furthermore, there are loci in chromosomes 1R and 7R that can increase wheat zinc efficiency [59] and loci in 5RL that increase copper efficiency [60]. Allelopathic effect on weeds is another favorable trait of rye chromatin in wheat, predominantly found in 1R and 2R substitutions [61].

## Conclusions

Plant breeders are continuously trying to find new sources for resistance that can be transferred into elite wheat germplasm. To find and transfer resistance genes that are effective against a wide range of strains of the key pathogen is ideal. Furthermore, it is desirable that those durable resistance genes are also effective against other pathogens, and other pests. Genes giving these plant characteristics have only been found in hexaploid wheat so far. For instance *Lr34* and *Lr67* leaf rust resistance genes provide pleiotropic effects on powdery mildew, yellow rust and stem rust [62–64]. However, the information we present here facilitates decision making in terms of combining resistances from different wheat-rye derived lines into a single wheat genotype. Primarily, we expect breeders to use existing rye introgressions in wheat. However, with improved materials and methods for making introgressions, and molecular tools for detection of rye chromatin in wheat, we expect also new introgression lines to be developed and used in wheat breeding.
